# Curated findings and implications in duplex ultrasound interrogation of the scrotum or varicoceles

**DOI:** 10.1038/s41598-020-78619-1

**Published:** 2020-12-16

**Authors:** Size Wu, Dongshen Zuo, Dongyan Cai, Qingfang Chen, Ya Li

**Affiliations:** Department of Ultrasound, The First Affiliated Hospital of Hainan Medical University, No. 31, Longhua Road, Haikou, 570102 China

**Keywords:** Diseases, Urology

## Abstract

The purpose of this study was to curate clustered findings of duplex ultrasound in the evaluation of spermatic venous varicoceles, and deliver more responses to the present concerns. Archives of 979 men who had undergone scrotum and spermatic venous plexus duplex ultrasound were reviewed. In the duplex ultrasound interrogation, the sizes of the larger vessels of the spermatic venous plexus, peritesticular vessels, and testicular volume and relevant parameters were measured. Findings of the vessels were analyzed. One hundred and eight-one out of 979 patients had varicoceles. Color Doppler flow signal was rendered in veins of pampiniform plexus but not in peritesticular vessels in 501 out of 979 patients; 101 out of 501 patients had veins of pampiniform plexus ≤ 3 mm, no color Doppler flow signal could be rendered in the veins in the 101 patients at supine and standing positions without Valsalva maneuver, color Doppler flow signal could be rendered in the veins in 82 out of 101 patients at supine and standing positions with Valsalva maneuver; no color Doppler flow signal could be rendered in the veins from 19 out of 101 patients with and without Valsalva maneuver at supine and standing positions. 37 out of 979 patients with 61 ipsilateral testicular volume ≤ 5 mL had no vessel diameter > 2 mm. The incidences of varicoceles corresponding to different ranges of testicular volume of 1–5 mL, 5.1–10 mL, 10.1–15 mL, 15.1–20 mL, 20.1–25 mL, and 25.1–30 mL were 0.0%, 6.9%, 8.3%, 6.63%, 20.94%, and 59.1%, respectively. The comparisons of incidences of varicocele between distribution percentages of different ranges of testicular volume of 1–5 mL and others (of 5.1 mL and more) were all significant (all *P* < 0.05). The correlation coefficient between the different ranges of testicular volume and the incidence of varicoceles was 0.829. Increased testicular volume may be also a factor for the development of varicoceles. Dilated peritesticular vessels may be collateral veins of spermatic veins, anterior and posterior scrotal veins, or proximal vas deferens.

## Introduction

Varicocele is an abnormal dilation of the pampiniform plexus of veins within the inguinal canal and scrotum, and it is a relatively common clinical problem, both in asymptomatic men and even more prevalent in subfertile men, representing the most common potentially treatable cause of male infertility^1^. Of varicocele, there has been an estimated prevalence of 15% in the general population, 35%-39% in primary male infertility^2,3^. Diagnosis of varicoceles depends on clinical scenarios, mainly the presence of dilated and tortuous veins in the spermatic cord, and medical imaging evaluation. In accordance to ultrasound evaluation of varicoceles: guidelines and recommendations of the European Society of Urogenital Radiology Scrotal and Penile Imaging Working Group (ESUR-SPIWG) for detection, classification, and grading, varicoceles is defined by the presence of multiple veins greater than 3.0 mm with concomitant retrograde blood^4,5^. Copious studies on diagnosis, grading, mechanism, and management of varicoceles have been published, many consensuses have achieved, however, some controversies and concerns remain unsolved^2–10^. The purpose of this study was to curate clustered findings of duplex ultrasound in the evaluation of the scrotum and spermatic venous varicoceles, and deliver more response to the present concerns.

## Materials and methods

### Study population

**Figure 1:**
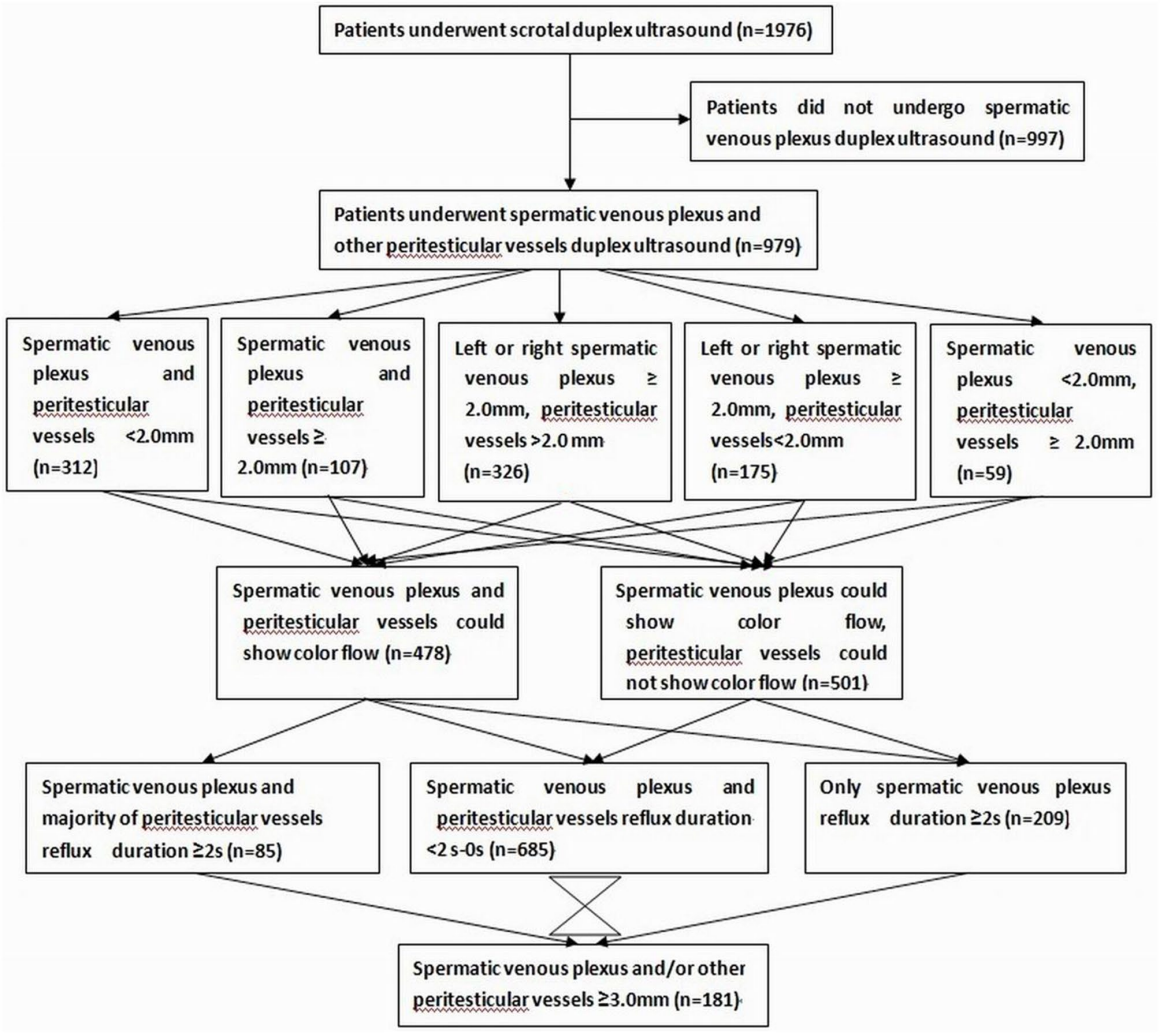
Flowchart of sample selection and duplex ultrasound interrogation.

Archives of 1976 men who had undergone consecutively scrotal duplex ultrasound evaluation in a tertiary hospital from January 1, 2016 to January 1, 2020 were reviewed. 979 men who had undergone scrotum and spermatic venous plexus duplex ultrasound for a clinically visualized venous plexus or collateral vein dilatation, palpable venous plexus enlargement, or spermatic cord fullness, which increased in size on assumption of the upright position or during the Valsalva maneuver, or other causes were included, and 997 patients who had spermatic cord hydrocele, hydrocele of tunica vaginalis testis, inguinal hernias, crytorchidism, testicular tumors, testicular torsion, epididymical lesions, epididymal agenesis, with history of varicocele repair, and who had not undergone spermatic venous plexus duplex ultrasound were excluded, as shown in Fig. 1. The age of enrolled patients ranged from 16 years old to 83 years old (the median age was 42 years old). The documents of spermatic venous plexus and other vessels in the scrotum saved in the Picture Archiving and Communication Systems (PACS) were studied retrospectively.

### Duplex ultrasound examination

The ultrasound systems used during this period included Hitachi-Aloka prosoundα7 (Hitachi Aloka Medical Systems, Tokyo, Japan); Mindray DC-8 (Shenzhen Mindray Bio-Medical Electronics Co., Ltd., Shenzhen, China); Mindray Resona 7 (Shenzhen Mindray Bio-Medical Electronics Co., Ltd., Shenzhen, China); Siemens Acuson S2000 (Siemens Medical Systems, Inc., Ultrasound Group, Mountain View, California, USA); Philips EPIQ5 (Philips Ultrasound, Inc., Bothell, Washington, USA); GE Logiq E9 (GE Healthcare, Waukesha, WI, USA). The patients were examined in the supine and standing position consecutively in the air-conditioned rooms with an ambient temperature of 24–30 °C. After the scrotum was exposed properly, adequate volume couple gel (heated to 33–35 °C) was placed onto the scrotum. The transducer was placed on the scrotum without exerting pressure. The linear-array transducers equipped in different ultrasound imaging systems with frequencies from 5 to 14 MHz were used to scan the scrotum, and multiple parameters were assessed, including bilateral testicular volume, echogenicity, echotexture, anatomic variants, abnormalities of the testes, epididymides, spermatic venous plexus vessels, peritesticular vessels, tunica vaginalis, inguinal canal, and scrotal wall. Color Doppler flow imaging and pulsed wave Doppler assessment were performed as an integral part of examination to detect and characterize venous reflux at the level of the inguinal canal, supratesticular region, and level of the testis. The sizes of the larger vessels were measured, irrespective of vessel location (within the inguinal canal or scrotum), patient position (supine or standing), or with and without Valsalva maneuver. In patients with an isolated right-sided varicocele, the abdomen was examined for abdominal and retroperitoneal pathology, as well as congenital vascular anomalies. For each examination, the examination mode was preset to “small parts and testis”, parameters of gain, depth gain compensate, focus, depth, and scale or pulse repetition frequency were adjusted to an adequate status. Images of duplex ultrasound imaging with and without measurements were saved in the PACS. The duplex ultrasound findings of spermatic venous plexus vessels, peritesticular vessels, epididymis, testis, and scrotum were analyzed.

### Statistical analysis

The testicular sizes were all re-calculated using up-to-date formula volume = Length × Height × Width × 0.71^4^. The incidences of varicoceles corresponding to different ranges of testicular volume of 1–5 mL, 5.1–10 mL, 10.1–15 mL, 15.1–20 mL, 20.1–25 mL, and 25.1–30 mL were calculated using percentile method. The incidences of varicocele between distribution percentages of testicular volume of 1–5 mL and others (of 5.1 mL and more) were compared using Chi-square analysis. Nonparametric correlations (spearman) were used to evaluate the relationship between the different ranges of testicular volume and the incidences of varicoceles. The level of statistical significance was set at *p* < 0.05 (2-tailed). Statistical analyses were performed with SPSS 20 (SPSS, IBM, Armonk, NY) statistical software.

### Ethical statements

All procedures followed were in accordance with the ethical standards of the responsible committee of the first affiliated hospital of Hainan medical university on human experimentation (institutional and national) and with the World Medical Association Declaration of Helsinki (revised in 2000). The study was approved by the institutional review board of the first affiliated hospital of Hainan medical university, and informed consent was waived due to the retrospective design.

## Results

**Figure 2:**
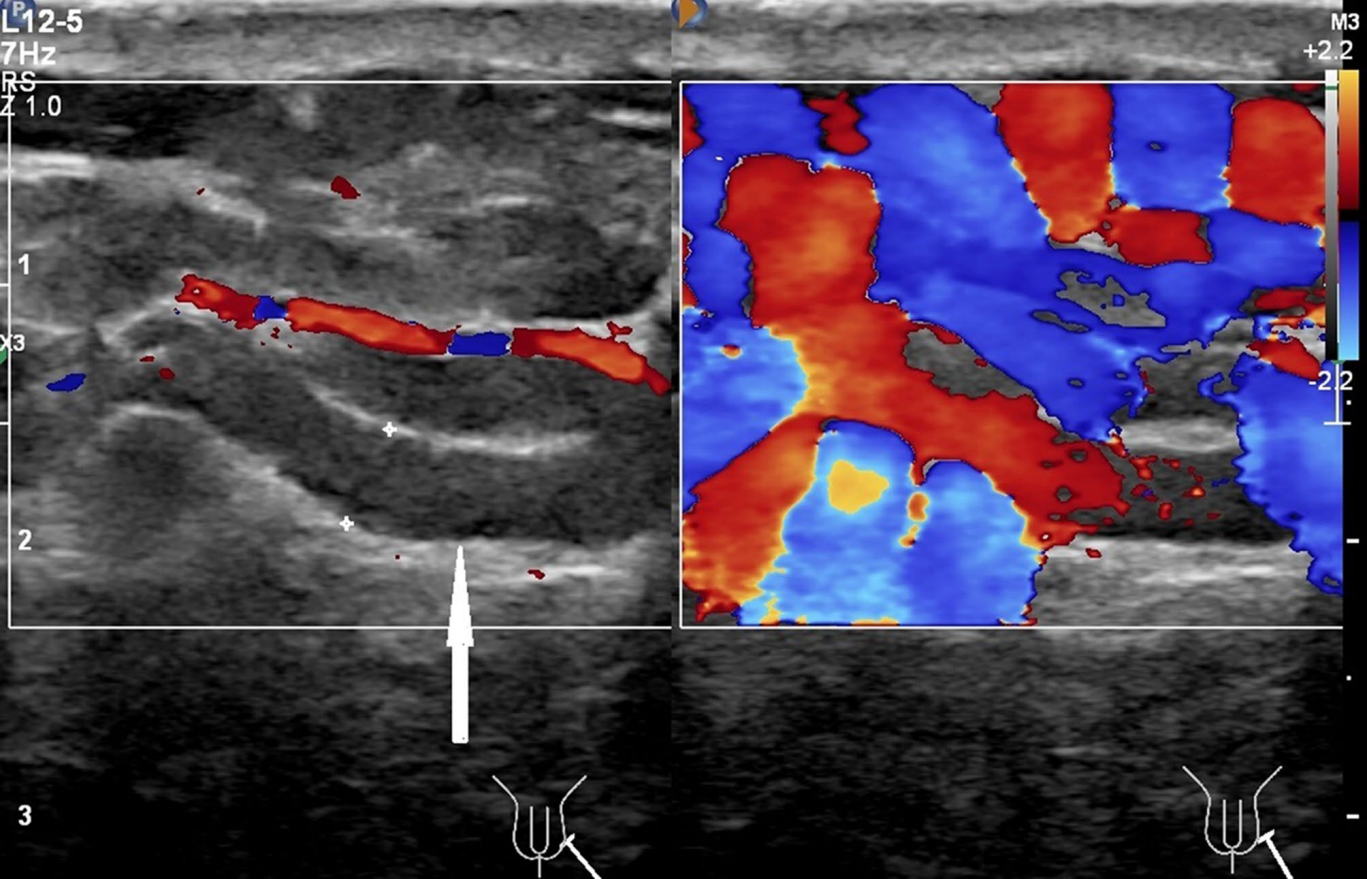
A 34-year-old man with left varicoceles. The veins of left pampiniform plexus present irregular serpiginous fluid-filled anechoic tubular structures (3.2 mm in diameter) in the region of the spermatic cord on gray-scale ultrasound at standing position (left panel, arrow); the fluid-filled anechoic tubular structures show color Doppler flow signal on duplex ultrasound (right panel).

One hundred and eight-one out of 979 patients had spermatic venous diameter > 3.0 mm. Of these dilated veins, 144 out of 181 veins located left side, nine out of 181 veins located right side, and 28 out of 181 veins located bilaterally. Color Doppler flow signal and reflux flow could be detected using duplex ultrasound in these veins, as shown in Fig. 2. Color Doppler flow signal was rendered in veins of pampiniform plexus but not in peritesticular vessels in 501 out of 979 patients; 101 out of 501 patients had veins of pampiniform plexus ≤ 3 mm, no color Doppler flow signal could be rendered in the veins in the 101 patients at supine and standing positions without Valsalva maneuver; color Doppler flow signal could be rendered in the veins in 82 out of 101 patients at supine and standing positions with Valsalva maneuver; no color Doppler flow signal could be rendered in the veins in 19 out of 101 patients with and without Valsalva maneuver at supine and standing positions. The flow reflux durations were not proportional to the sizes of veins. Venous diameters of pampiniform plexus and some peritesticular vessels had a little increase in the standing position and / or during the Valsalva maneuver in major patients. In 37 out of 979 patients with 61 ipsilateral testicular volume < 5 mL, the venous diameters of pampiniform plexus and peritesticular vessels were all < 2 mm. Venous diameters of pampiniform plexus in 117 out of 979 patients with 213 ipsilateral testicular volume > 20 mL were all > 2.0 mm. No intratesticular varicocele was found in 979 patients. Tubular ectasia of the rete testis was found in two out of the 181 patients with varicoceles. The incidences of varicoceles corresponding to different ranges of testicular volume of 1–5 mL, 5.1–10 mL, 10.1–15 mL, 15.1–20 mL, 20.1–25 mL, and 25.1–30 mL were 0.0%, 6.9%, 8.3%, 6.63%, 20.94%, and 59.1%, respectively. The comparisons of incidences of varicocele between distribution percentages of different ranges of testicular volume of 1–5 mL and others (of 5.1 mL and more) were all significant (all *P* < 0.05). The correlation coefficient between the different ranges of testicular volume and the incidence of varicoceles was 0.829 (*P* = 0.042). Distributions of testicular volumes, maximal venous diameters of pampiniform plexus and percentiles of varicoceles were listed in Table 1. Findings of spermatic venous plexus and other scrotal vessels assessed by duplex ultrasound were shown in Fig. 1.

**Table 1:** Distributions of testicular volumes, maximal venous diameters of pampiniform plexus and percentile of varicoceles.

Testicular volume (mL) and testicles	Maximal venous diameters of pampiniform plexus (mm)							Percentile of varicoceles (%)
	0.1–1.0	1.1–2.0	2.1–3.0	3.1–4.0	4.1–5.0	5.1–6.0	6.1–7.0	
1.0–5.0 (n = 61)	52	9	0	0	0	0	0	0.0
5.1–10.0 (n = 319)	62	199	36	22	0	0	0	6.9
10.1–15.0(n = 798)	7	504	221	55	7	3	1	8.3
15.1–20.0 (n = 603)	0	330	233	36	4	0	0	6.6
20.1–25.0 (n = 191)	0	17	134	35	3	2	0	20.9
25.1–30.0 (n = 22)	0	3	6	12	1	0	0	59.1
Total (n = 1994)	121	1062	630	160	15	5	1	9.1

## Discussion

In our series of 979 patients undergoing scrotal evaluation, 181 patients with varicoceles (≥ 3 mm) had not referred to physician before, accounting for 18.5% (181/979) of all evaluated patients. The reason is that majority of them had mild symptoms, and they did not pay much attention to the symptoms and / or were not aware of the underlying disease. The main cause and mechanism of varicoceles of pampiniform plexus has been interpreted as that the right testicular vein usually drains into the inferior vena cava while the left testicular vein drains into the left renal vein and gets confluence with the renal venous flow, then drains into the inferior vena cava. This anatomical difference induces relatively weak hemodynamics in the left testicular vein, which accounts for the predominance of left sided varicocele^6,7^ . Other causes include that the incompetence of venous valves results in reflux of venous blood and increases hydrostatic pressure; "nutcracker" phenomenon that the proximal left renal vein decreases with decrease in the aortomesenteric distance and angle; renal tumor compression, and so on^6–9^. In addition, we believe that the common presence of anatomical variations of testicular vessels and relevant vessels may be also the cause of varicoceles^10–15^. For if the distribution of testicular drainage in different veins is not appropriate, the spermatic vein drainage and hemodynamics may be affected, and these may cause left side, or right side varicoceles, or bilateral varicoceles.

In our study, no ipsilateral varicocele occurred in 37 adult patients with 61 ipsilateral testicular volume < 5 mL (18 patients with Klinefelter syndrome, 11 patients with mumps related orchitis, 8 patients with other diseases), and no diameter of pampiniform plexus was < 2.0 mm in patient with ipsilateral testicular volume > 20 mL. The comparison of incidences of varicocele between distribution percentages of different ranges of testicular volume of 1–5 mL and others (of 5.1 mL and more) were all significant (all *P* < 0.05), and the relationship between the different ranges of testicular volumes and the incidences of varicoceles was strong (correlation coefficient of 0.829). These indicate that the volume of testicular drainage associates with the testicular volume, the larger the testicular volume the more the drainage takes place. If the drainage does not match the arterial perfusion exactly, it may develop blood stasis and result in varicoceles.

In this study, color Doppler flow signal and flow reflux could be rendered in all vessels > 3 mm, while color Doppler flow signal and/ or flow reflux could only partially be rendered in vessels < 3 mm; and apparent flow reflux (> 2 s) could be rendered in normal size veins of pampiniform plexus and some small vessels (diameter ≤ 2 mm) of pampiniform plexus and peritesticular veins. These indicate that flow reflux presents commonly in veins with dilated diameter, and also presents in veins with normal diameter, while veins with diameter > 3 mm (varicoceles) are almost definitely abnormal. So we believe that dilated venous diameter is the fundamental parameter of varicoceles, and flow reflux is the second; although the duration of reflux time at Valsalva maneuver, an important parameter of varicoceles, can be used for varicoceles grading^4,7^.

**Figure 3:**
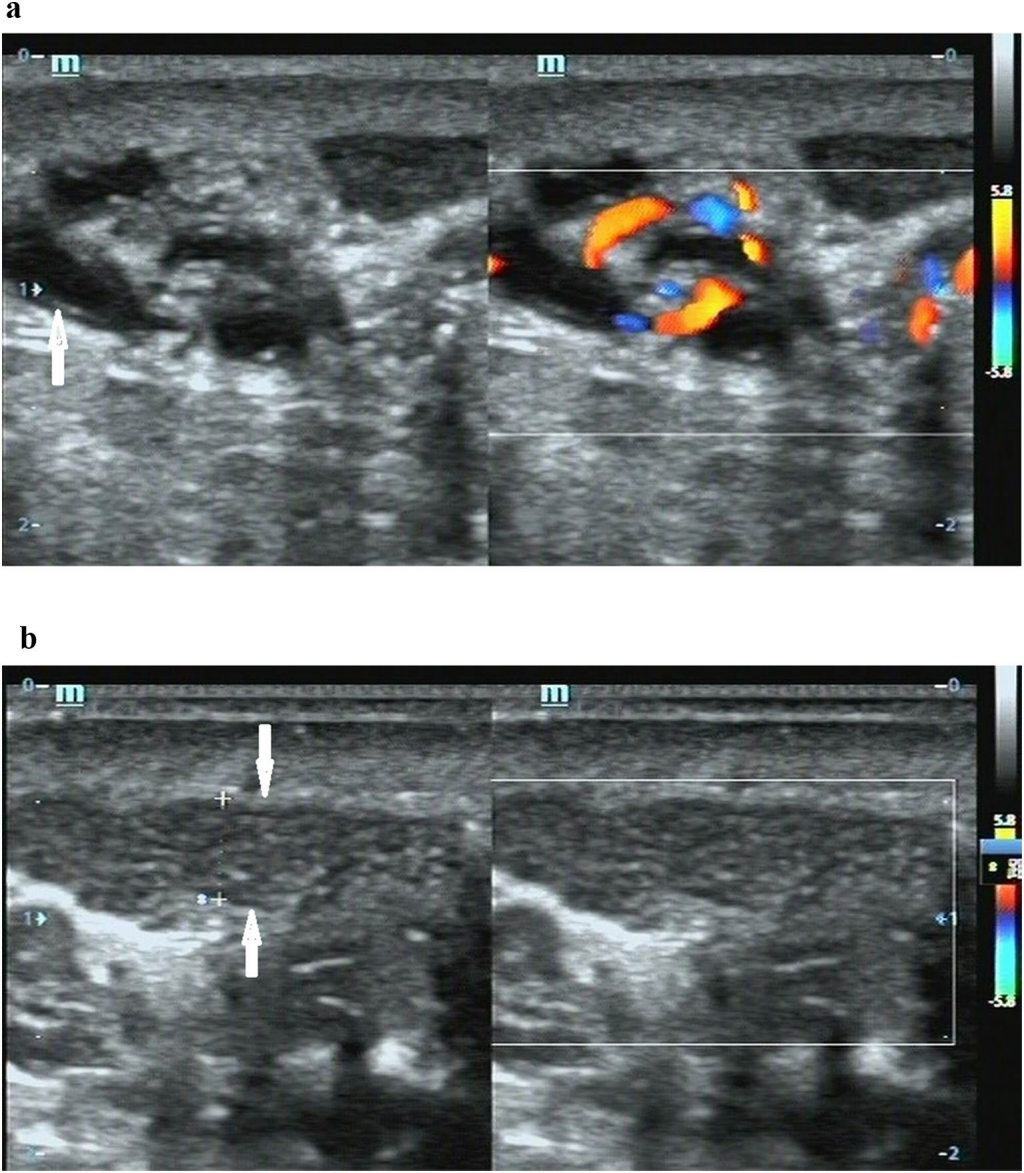
A 37-year-old man with dilated proximal vas deferens. The dilated proximal vas deferens present fluid-filled anechoic tubular structures without color Doppler flow signal in the region of the supratesticular region on duplex ultrasound at standing position with Valsalva maneuver, and the wall of the tubular structures is thicker than that of the pampiniform vein (arrow, **a**); the left epididymis present multiple thin tubular structures without color Doppler flow signal (arrows, **b**).

**Figure 4:**
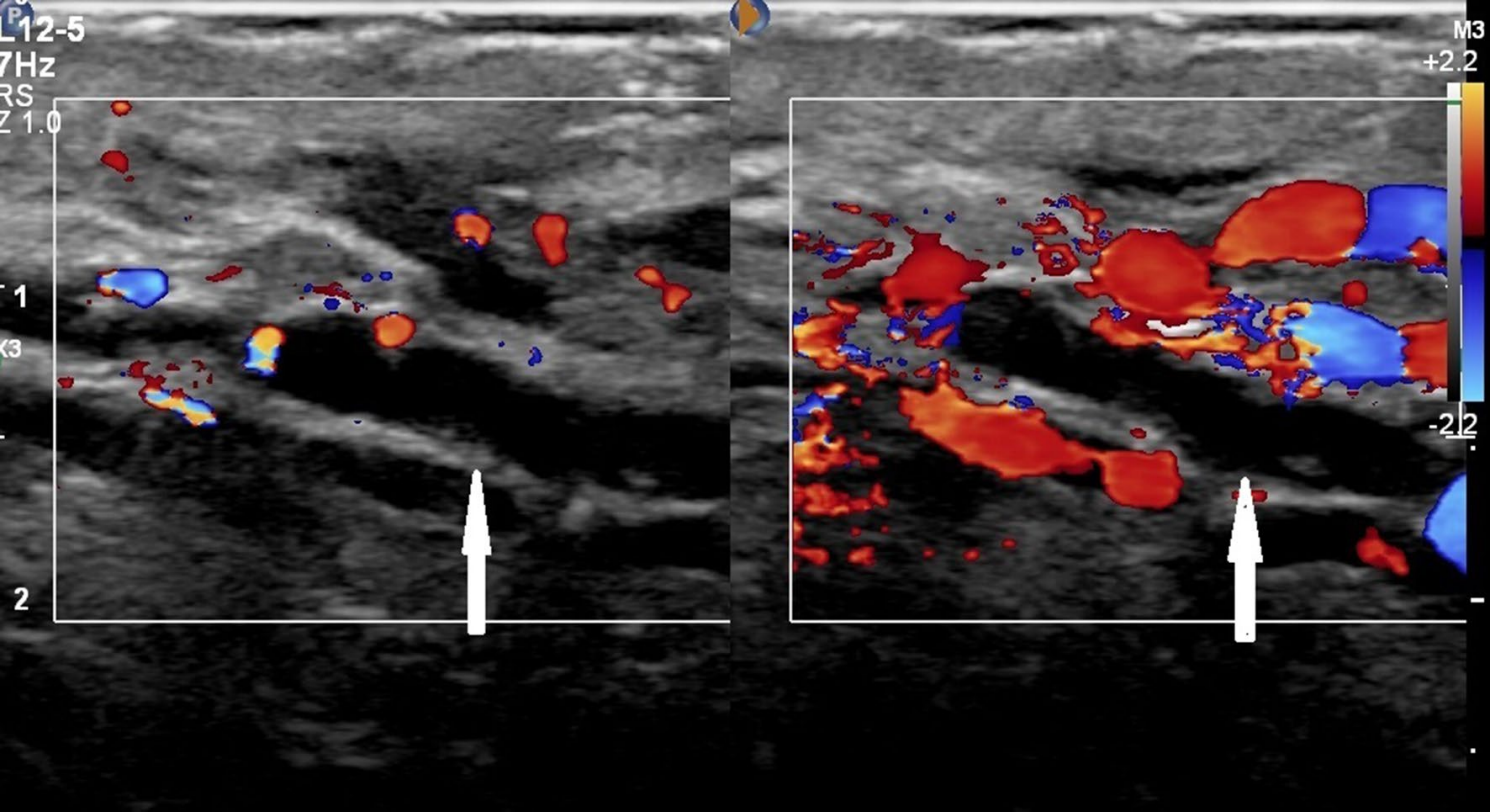
A 28-year-old man with dilated peritesticular veins. The veins of left pampiniform plexus present fluid-filled anechoic tubular structures in the region of the spermatic cord on duplex ultrasound at supine position without Valsalva maneuver (arrow, left panel); some of the fluid-filled anechoic tubular structures show color Doppler flow signal on duplex ultrasound at standing position with Valsalva maneuver, and the others do not show color Doppler flow signal (right panel). The scrotum does not show edematous and thicken.

There are 630 out of 979 patients with diameter of scrotal vessel ≤ 3 mm in this series. The profile of dilated scrotal peritesticular vessels may be either subclinical varicoceles according to the present diagnostic criteria of varicoceles, or collateral vein dilatation or other entities. Proximal vas deferens can be traced originating from the epididymis, the wall of it is thicker than vein, and it is recognizable (as shown on Fig. 3). If the lymphatic vessels dilate, the scrotal wall will be edematous and thicken, and it can be identified. If there is color Doppler flow signal in the vessel, the vessel may be a venous tributary of different veins; if there is no color Doppler flow signal in the vessel, the vessel may be a collateral vein of spermatic vein with blood stasis (as shown on Fig. 4) or a vein of scrotal wall. The reasons that color Doppler flow signal cannot be rendered in the blood vessels may be that the blood in the vessels is of stasis or the velocity is very low, and the blood vessels do not subject to supine and standing changes of body position and / or Valsalva maneuver. Different from drainage to inferior vena cava and proximal renal vein that is ready to be affected by supine and standing changes of body position and/or Valsalva maneuver, drainages of external pudendal vein, cremasteric veins and some anastomoses of pampiniform venous plexus with anatomical variation are usually not affected by supine and standing changes of body position and/or Valsalva maneuver. As have been addressed in the studies that the route of venous drainage of the testis was the internal spermatic vein and the external pudendal vein; some venous drainage of the testis has communication with the vein of epididymis, deferential veins, cremasteric veins (anterior and posterior scrotal veins), ureteral veins, colonic and renal capsular veins^10,16–18^. Tubular ectasia of the rete testis is a rare vessel entity that can be encountered occasionally in the scrotal evaluation^19^. Tubular ectasia of the rete testis was found in two of the 181 patients with varicoceles in this study, and no color Doppler flow signal was rendered from them. Tubular ectasia of the rete testis occurs in the testis, which is different from intratesticular varicoceles, and it does not subject to the dilated vein of pampiniform plexus.

The potential limitations of the present study were that the assessment and measurement were performed by different physicians on different ultrasound imaging systems, the interobserver agreement had not been evaluated, which might cause errors in some extent. However, our previous studies and experiences had shown that the measurements of vessels on high frequency ultrasonography and at zoom mode are reproducible, which causes only a little error, so it may be overlooked. The other caveats are that the drainage route of spermatic veins had not been tracked, and the specific causes and mechanism for varicoceles were not validated and further understood.

## Conclusions

Spermatic venous varicoceles and dilated peritesticular vessels present various manifestations at duplex ultrasound imaging interrogation. The relationship between the different ranges of testicular volume and the incidences of varicoceles was strong, and varicoceles had not found in spermatic veins of ipsilateral testicular volume ≤ 5 mL, which indicate that increased testicular volume may be also a factor for the development of varicoceles. Venous diameter > 3.0 mm is the fundamental criterium of varicoceles, and flow reflux is the second. The dilated peritesticular vessels include collateral veins of spermatic cord veins, anterior and posterior scrotal veins, or proximal vas deferens (Suppl Information).

## Supplementary Information

Supplementary Legends.

Supplementary Figure S1.

Supplementary Figure S2.

Supplementary Figure S3.

Supplementary Figure S4.

Supplementary Figure S5.

Supplementary Figure S6A.

Supplementary Figure S6B.
