# Supplementary material for: Curated findings and implications in duplex ultrasound interrogation of the scrotum or varicoceles
Source: Sci Rep. 2020 Dec 16;10:22028. doi: 10.1038/s41598-020-78619-1 (PMC7744525; doi:10.1038/s41598-020-78619-1)
Supplement: Supplementary file 1 — Supplementary Legends. [file 41598_2020_78619_MOESM1_ESM.docx]

**Curated findings and implications in duplex ultrasound interrogation of the scrotum or varicoceles**

*Size Wu, Dongsheng Zuo, Dongyan Cai, Qingfang Chen, Ya Li*

**Legends for figures**

Figure 1 A 26-year-old man with mild scrotum pain and palpable enlarged left spermatic cord. Ultrasonography obtained at standing position and Valsalva maneuver shows enlarged veins of left spermatic plexus, with one vein measured 6.1mm in diameter, indicating varicoceles.

Figure 2 A 32-year-old infertile man without uncomfortable scrotum. Duplex ultrasound obtained at standing position and Valsalva maneuver shows enlarged veins of left spermatic plexus, with one vein measured 2.7mm in diameter, indicating subclinical varicoceles.

Figure 3 A 21-year-old man with uncomfortable scrotum. Duplex ultrasound shows vessels structure at the root of left scrotum obtained at standing position and Valsalva maneuver, with one vessel without color Doppler flow imaging measured 2.2mm in diameter, indicating it may not be a vein of pampiniform plexus, and it may be a peritesticular vein with static blood.

Figure 4 A 32-year-old infertile man without uncomfortable scrotum. Duplex ultrasound image obtained at standing position and Valsalva maneuver at the left root of scrotum, detected using pulsed wave Doppler shows longer reflux duration of a dilated vein of pampiniform plexus.

Figure 5 A 22-year-old man with uncomfortable scrotum. Ultrasound measurement of the testicular volume using the built-in software of the ultrasound system shows the volume is 18.4mL, indicating the formula is that volume = Length × Height × Width × 0.52. Recalculated the original measurements manually using formula of volume = Length × Height × Width × 0.71, the volume is 24.9mL.

Figure 6a and b A 30-year-old infertile man without uncomfortable scrotum. Duplex ultrasound shows his two testes are of microlithiasis, and the testicular volume is well below the standard volume (20mL). His veins of pampiniform plexus are smaller than 1.0mm (not shown).
