# Supplementary figures and images for: Curated findings and implications in duplex ultrasound interrogation of the scrotum or varicoceles
Source: Sci Rep. 2020 Dec 16;10:22028. doi: 10.1038/s41598-020-78619-1 (PMC7744525; doi:10.1038/s41598-020-78619-1)

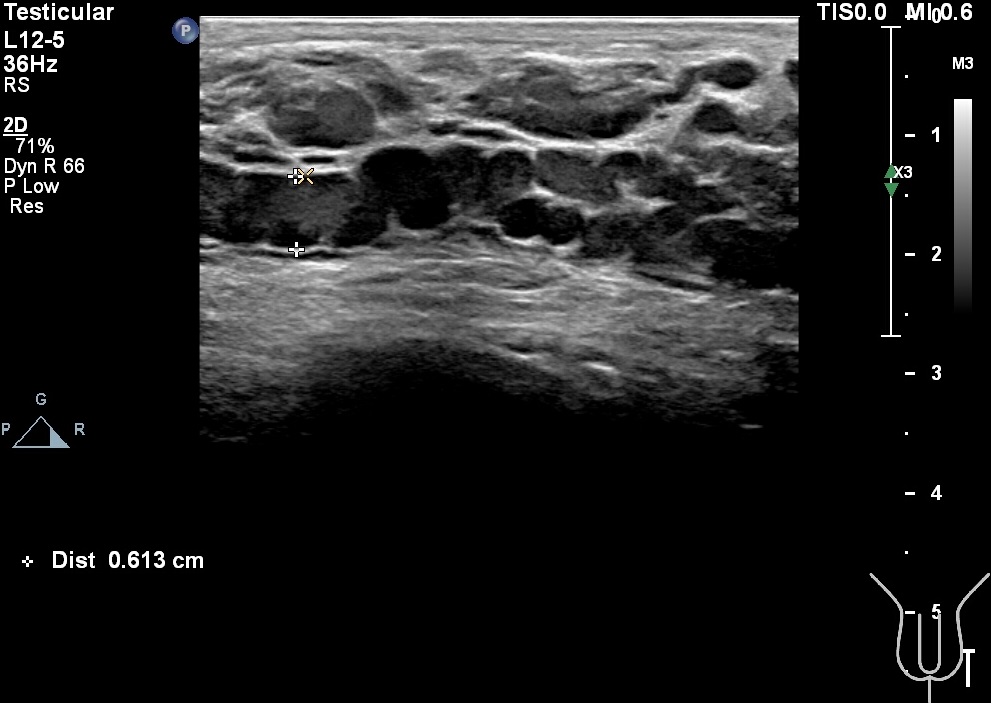

Supplement: Supplementary file 2 — Supplementary Figure S1. [file 41598_2020_78619_MOESM2_ESM.jpg]

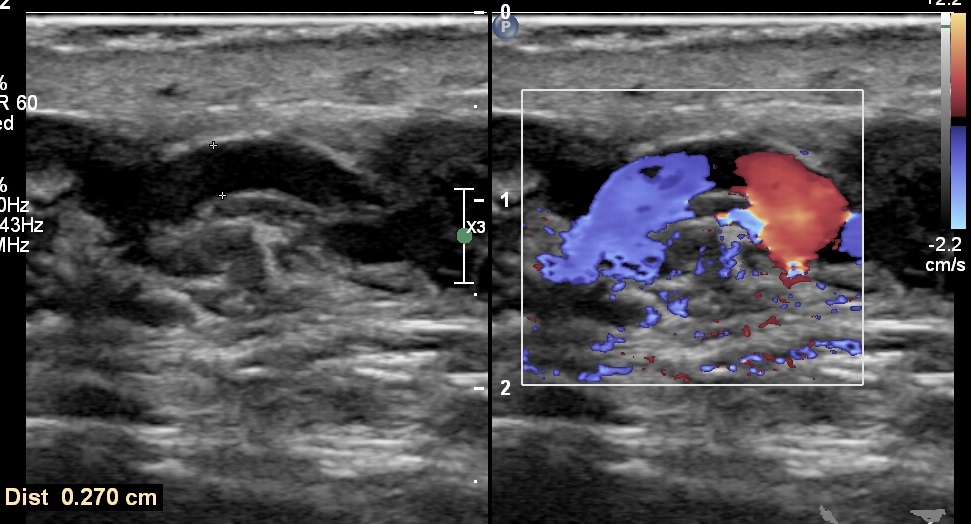

Supplement: Supplementary file 3 — Supplementary Figure S2. [file 41598_2020_78619_MOESM3_ESM.jpg]

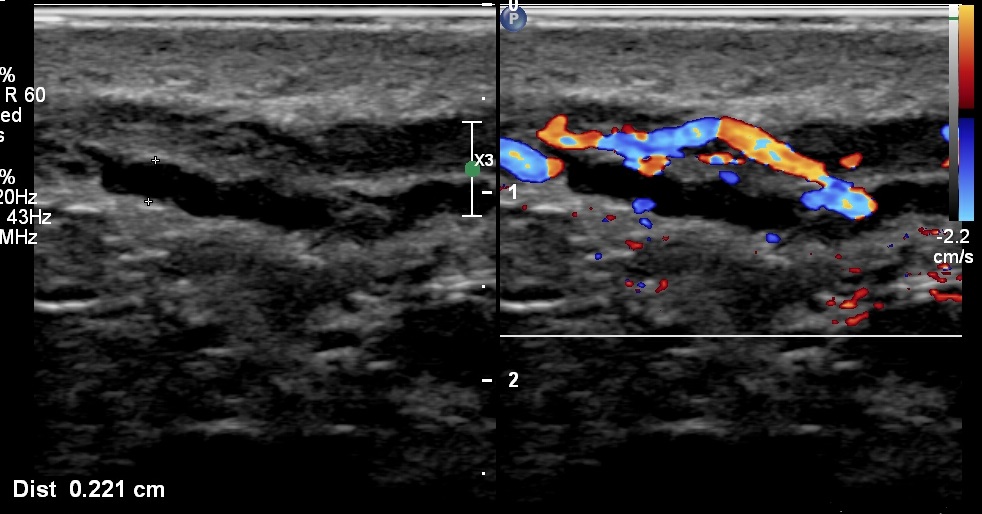

Supplement: Supplementary file 4 — Supplementary Figure S3. [file 41598_2020_78619_MOESM4_ESM.jpg]

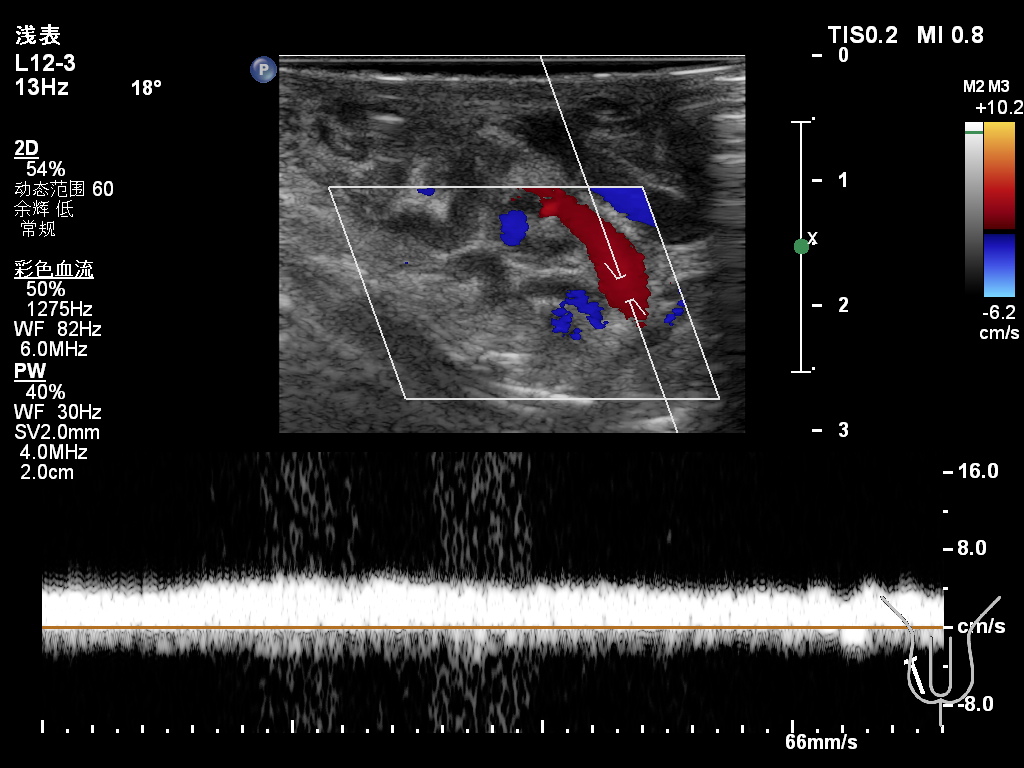

Supplement: Supplementary file 5 — Supplementary Figure S4. [file 41598_2020_78619_MOESM5_ESM.jpg]

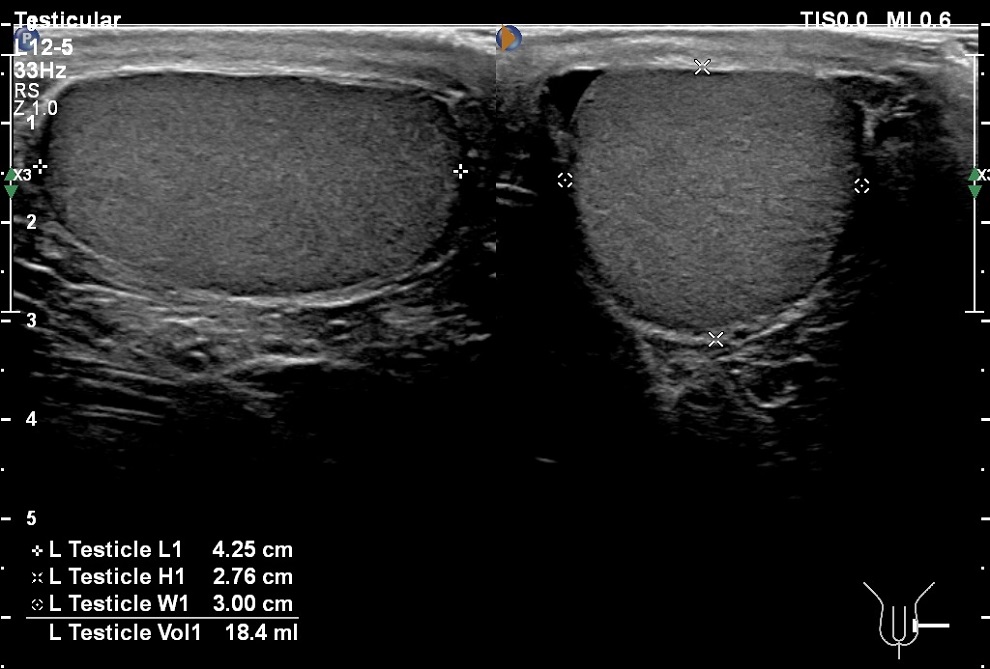

Supplement: Supplementary file 6 — Supplementary Figure S5. [file 41598_2020_78619_MOESM6_ESM.jpg]

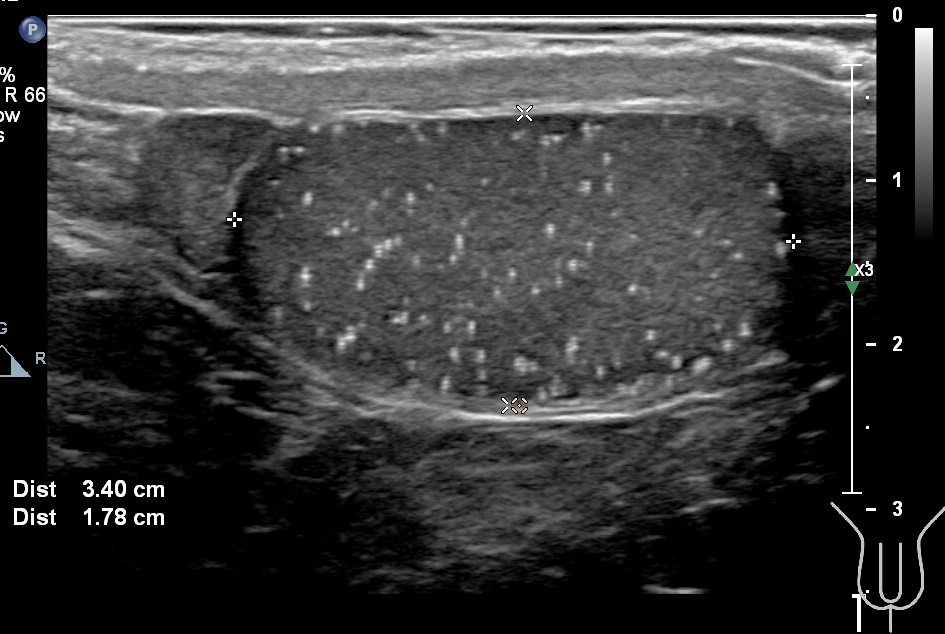

Supplement: Supplementary file 7 — Supplementary Figure S6A. [file 41598_2020_78619_MOESM7_ESM.jpg]

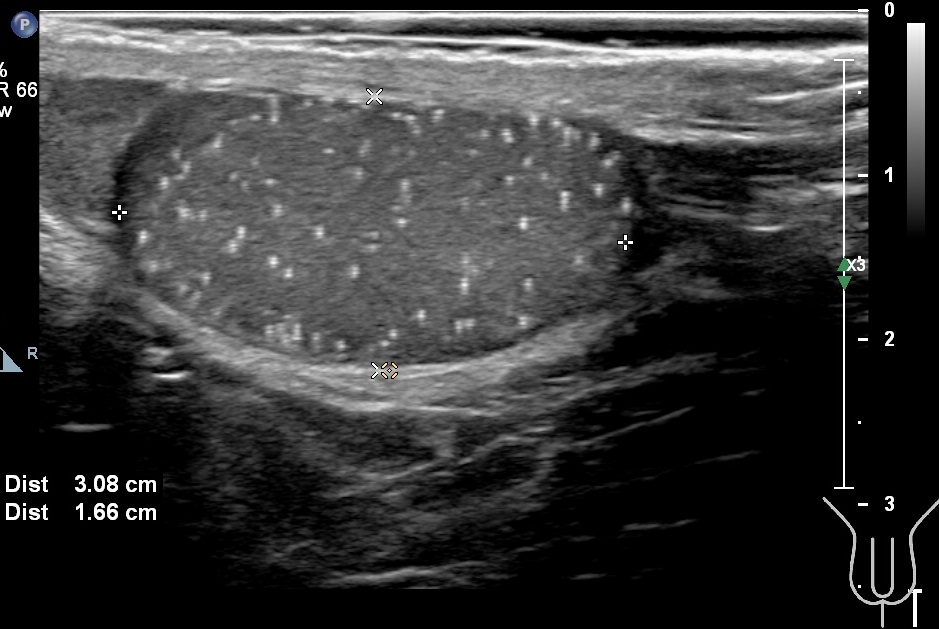

Supplement: Supplementary file 8 — Supplementary Figure S6B. [file 41598_2020_78619_MOESM8_ESM.jpg]
